# Transfer learned deep feature based crack detection using support vector machine: a comparative study

**DOI:** 10.1038/s41598-024-63767-5

**Published:** 2024-06-24

**Authors:** K. S. Bhalaji Kharthik, Edeh Michael Onyema, Saurav Mallik, B. V. V. Siva Prasad, Hong Qin, C. Selvi, O. K. Sikha

**Affiliations:** 1 Department of Computer Science and Engineering, Amrita School of Computing, Amrita Vishwa Vidyapeetham, Coimbatore, Tamil Nadu 641112 India; 2 Department of Mathematics and Computer Science, Coal City University, Enugu, Nigeria; 3 Adjunct Faculty, Saveetha School of Engineering, Saveetha Institute of Medical and Technical Sciences, Chennai, India; 4 Department of Environmental Health, Harvard T H Chan School of Public Health, Boston, MA 02115 USA; 5 School of Engineering (CSE), Anurag University, Hyderabad, India; 6 Department of Computer Science and Engineering, The University of Tennessee at Chattanooga, Chattanooga, TN USA; 7 Department of Computer Science and Engineering, Indian Institute of Information Technology, Kottayam, Kerala 686635 India; 8 Dept. of Information and Communication Technologies, BCN Medtech, Universitat Pompeu Fabra, Barcelona, Spain

**Keywords:** Convolutional neural networks, Crack detection, Support vector machine (SVM), Transfer learning, Ecology, Environmental sciences, Engineering, Mathematics and computing

## Abstract

Technology offers a lot of potential that is being used to improve the integrity and efficiency of infrastructures. Crack is one of the major concerns that can affect the integrity or usability of any structure. Oftentimes, the use of manual inspection methods leads to delays which can worsen the situation. Automated crack detection has become very necessary for efficient management and inspection of critical infrastructures. Previous research in crack detection employed classification and localization-based models using Deep Convolutional Neural Networks (DCNNs). This study suggests and compares the effectiveness of transfer learned DCNNs for crack detection as a classification model and as a feature extractor to overcome this restriction. The main objective of this paper is to present various methods of crack detection on surfaces and compare their performance over 3 different datasets. Experiments conducted in this work are threefold: initially, the effectiveness of 12 transfer learned DCNN models for crack detection is analyzed on three publicly available datasets: SDNET, CCIC and BSD. With an accuracy of 53.40%, ResNet101 outperformed other models on the SDNET dataset. EfficientNetB0 was the most accurate (98.8%) model on the BSD dataset, and ResNet50 performed better with an accuracy of 99.8% on the CCIC dataset. Secondly, two image enhancement methods are employed to enhance the images and are transferred learned on the 12 DCNNs in pursuance of improving the performance of the SDNET dataset. The results from the experiments show that the enhanced images improved the accuracy of transfer-learned crack detection models significantly. Furthermore, deep features extracted from the last fully connected layer of the DCNNs are used to train the Support Vector Machine (SVM). The integration of deep features with SVM enhanced the detection accuracy across all the DCNN-dataset combinations, according to analysis in terms of accuracy, precision, recall, and F1-score.

## Introduction

Cracks in concrete structures, resulting from factors like rust, chemical degradation, and unfavorable loading, serve as warning signs for tension, fragility, and wear. The length, width, depth, and position of these cracks impact their significance^1^. To ensure the long-term serviceability of infrastructures, monitoring structural health and performance is crucial^2^. Traditional manual inspection methods relying on eyesight are time-consuming, labor-intensive, and prone to subjective conclusions. The high cost of labor and potential human error makes frequent manual inspections impractical. Efficiently identifying surface cracks within a specific timeframe is crucial for enhancing the maintenance protocols of buildings. This swift detection allows for timely interventions, preventing the deterioration of structural issues and minimizing repair costs. By promptly addressing these cracks, potential safety hazards can be mitigated, ensuring the longevity and structural integrity of the building. Recent advancements in science and technology have led to the development of automatic crack detection models, employing image processing and machine learning (ML) techniques^3–6^.

Image processing-based techniques use statistical features from structural images to detect and locate cracks, treating them as regions with sudden pixel intensity changes. Machine Learning (ML)-based models utilize hand-crafted features, such as edge, texture, and color, for automatic crack detection^7,8^. With the availability of massive datasets, researchers have turned to Deep Learning (DL), particularly Convolutional Neural Networks (CNN), for more effective crack detection. The success of DL-based models, especially neural networks with multiple layers, has significantly improved feature learning. CNNs, with varied filters highlighting crucial features, extract basic image features in initial layers and advanced, crack-specific features in deeper layers. These features are then passed to a multi-layer perceptron classifier for crack detection. The accessibility of powerful computing resources and continuous advancements in training techniques on readily available datasets propel the rapid development of deep learning. Despite the success in feature extraction, there’s a need to enhance the accuracy of these models in detecting concrete cracks.

In this research, we put forth a method of transfer learning-based deep convolutional neural networks (DCNN) with the pre-trained weights as a classifier and feature extractor, which exhibits a considerable increase in terms of performance, unavailability of large dataset and training time. This paper also investigates the impact of ML classifiers learned over deep features for crack detection. Three publicly available datasets were used for the study SDNET2018^9^, Concrete Crack Images for Classification (CCIC)^10^, and Bridge Crack Dataset (BCD)^11^. Experiments conducted in this work are threefold (1) Crack detection based on transfer learned deep CNNs: 12 state-of-the-art CNN models transfer learned on ImageNet were used to classify the crack images (2) Crack detection using transfer learned CNNs on enhanced crack images (3) Examining DCNN’s performance as a feature extractor.

The obtained features from deep CNNs’ fully connected layers (final FC layers) are classified and compared using ML algorithms. The major contributions of the proposed work are:

1. Classification of crack images using 12 transfer-learned DCNNs including VGG16, VGG19, Xception, ResNet50, ResNet101, ResNet152, InceptionV3, InceptionResNetV2, MobileNet, MobileNetV2, DenseNet121 and EfficientNetB0.
2. Analysis of the effectiveness of image enhancement techniques such as contrast enhancement and Local Binary Pattern (LBP) pre-processing on transfer learned DCNN models for crack detection.
3. Development of Support Vector Machine (SVM)-ML-based classification model on deep features extracted from the aforementioned DCNN models.

The following is how the paper is organized: The related crack detection research is covered in Section “Literature review**”** of this paper. The proposed system and the experiments carried out to categorize the images are described in Section “Proposed Methodology”. A description of the various datasets used is provided in Section “Dataset”. The outcomes and conclusions of the experiment are described in Section “Experimental Result and Analysis”. The paper is concluded in Section “Conclusion and Future Scope”.

## Literature review

A thorough description of the most recent crack detection models is provided in this section. Crack detection models found in the literature can be divided into three major categories based on their workflow: (1) Models based on traditional image processing algorithms (2) Models based on machine learning models (3) Models based on deep learning models.

### Classical image processing-based models

Crack detection using image processing methods have three major steps: image acquisition, pre-processing, and crack detection^12^. The target component is first photographed in high quality with a camera or any other imaging instrument. The next step in the pre-processing is to eliminate noise and shadows from the images by applying filters, segmentation, and other techniques. If necessary for the particular crack detection technique being used, the image may be transformed to gray-scale or binary format. The generated image is then put through crack detection, which emphasizes or segments the image’s cracked area using image processing techniques like edge detection, segmentation, or pixel analysis.^13^. Lins et al.^14^, developed a method to identify cracks using several color models like HSV (Hue-Saturation-Value) and RGB (Red–Green–Blue). They proposed a color feature extraction model, which searches for certain color compositions in an image in comparison to a standard query color. Further, the authors have used their crack measurement algorithm to measure the length and width of the detected cracks. Shahrokhinasab et al.^15^, analyzed various image processing methods like edge detection, and thresholding, to classify cracks. Munawar et al.^16^, analyzed different methods of fissure detection including genetic programming, beamlet transformation, Unmanned Aerial System based approach, and the Shi Tomasi algorithm. Zou et al.^17^, introduced an automated crack detection system titled as CrackTree which uses a geodesic shadow removal algorithm to eliminate shadows from pavement images.

A crack probability map is produced using tensor voting, and a graph model is built by choosing crack seeds from the crack probability map. Recursive edge pruning in the graph’s Minimum Spanning Tree (MST) is used to find the final crack curves. Gabor Filters were employed by Salman et al.^18^, for crack detection. Niu et al.^19^, introduced a method to find cracks in tunnels that involve a series of image processing, image filtering, and image feature extraction methods. They have used uniform light processing for the crack to appear better, used median and bilateral filtering to filter out the noise, and used a combination of Gabor filter and EMAP to extract required features. The features were then fed into the CEM algorithm to detect the cracks. Oliviera et al.^20^, used a group of pixel-based and block-based image processing algorithms. The image processing techniques used were anisotropic diffusion, Perona and Malik’s algorithm, morphological smoothening, alternative sequential filtering, a combination of morphological erosion and dilation operators, Symlet decomposition filters, and UINTA and R- UINTA. Baltazart et al.^21^, presented an improved version of the Minimum Spanning Tree algorithm to identify cracks called the MPS– VI and analyzed the computational time of each model. An ACDS architecture was proposed by Jo et al.^22^, which had an image acquisition block, a pre-processing block and the classification block. In the pre-processing block, they used the Hessian-based method, Gabor filter, Otsu, Retinex filter, and Median filter to extract features and use these features to train and classify the deep belief network. Classical image processing-based models for crack detection depend on the quality of images.

### Machine learning-based models

ML-based models for crack detection follow five steps: dataset collection, pre-processing of images, feature extraction, model training on the extracted features, and testing. Landstrom and Thurley^23^ employed morphological operators to slice the cracks from the image and logistic regression is used to distinguish the crack/non-crack images using the segmented images. Prasanna et al.^24^, put forth a crack detection method called spatially tuned robust multi-feature (STRUM), in which the authors have explored classifiers including SVM, AdaBoost, and Random Forest. Lin et al.^25^, used hidden Markov random field-expectation–maximization (HMRF–EM) for automatic pavement crack detection, with 2 major modules. Firstly, the hidden Markov random field model and its expectation–maximization are combined with the adaptive line detector to increase detecting accuracy. Secondly, the integrity and continuity of the detected cracks are improved by the quantitative description of the crack region’s credibility and conditional connection. FG Pratico et al.^26^, provided a method for classifying the structural health condition of several vibro-acoustically different road pavement cracks (concealed bottom-up cracks) using supervised machine learning techniques. The technique intends to gather the signatures (using roadside acoustic sensors) and categorize the structural health status of the pavement using ML models. They compared various ML classifiers, including the random forest classifier (RFC), support vector classifier (SVC) and multi-layer perceptron (MLP). Results indicate the SVC is the best-performing ML model with an accuracy of 99.1%. Zhang et al.^27^, suggested a new method for identifying surface fractures in coal mining sites using Unmanned Aerial Vehicle(UAV) imagery and ML.

The overall accuracy was increased to 88.99% by applying the V-SVM classifier. The authors also used Laplace sharpening to improve the color of the images and Principal Component Analysis (PCA) to minimize the entire set of features to 95% of the initial variance. A ML-computer vision pipeline was proposed by Zhang et al.^28^ for detecting the formation of fatigue cracks. Cracks were detected using an ML model, and vision-based algorithms were further utilized to examine the growth direction and length of the fatigue crack. The primary problem with ML-based models for crack detection is the selection and extraction of relevant features for the classifier’s training.

### Deep learning-based models

Numerous crack detection models have been developed in the literature as a result of recent developments in deep learning (DL), particularly the evolution of convolutional neural networks (CNNs)^29^. DL-based models for crack detection follow steps analogous to ML-based models described above. The major difference is that DL models do feature extraction implicitly. A dataset of surface cracks must be gathered first to train the DL model. To minimize noise, eliminate shadows, and modify other features like image size and brightness, the images are then pre-processed using image processing techniques. These images are then subjected to pixel-by-pixel annotation, or labeling, where the pixels corresponding to cracks are annotated either manually or by using annotation tools. One example of labeling is making the remaining pixels in the image black or “0” and the crack pixels white or “1” in the image. Following this, a DL architecture CNNS must be chosen to be applied to crack detection. Li et al.^30^, proposed a deep neural architecture with a convolutional block, four dense connections, five deep supervision modules, three conversion modules and one fusion module to identify cracked surfaces. Zhang et al.^31^, introduced a CNN architecture with four convolutional layers and two fully connected layers. Their convolution network achieved a precision of 0.869 and a recall of 0.925 for crack detection. Meng et al.^32^, proposed a deep residual neural network-based concrete crack identification method that identified concrete crack images at the pixel level. Transfer learned EfficientNetB0 was employed by C.Su and W. Wang^33^ for crack detection. They reported an accuracy better than that of a fully convolutional network proposed by Ye et al.^34^, which gave an accuracy of 93.6%. Feng et al.^35^, used transfer learning on the InceptionV3 model to classify cracks which included crack, intact, spalling, seepage and rebar exposure as the classes. A custom convolutional neural network with three convolutional layers was introduced by Kim et al.^36^ for crack detection. The images were pre-processed using morphological filters and contrast enhancement operators, which in turn were used to train the CNN model for the identification of cracks. Cao et al.^37^, used object-detecting paradigms such as faster RCNN and SSD models along with MobileNet, Inception, Resnet, and Inception Resnet to detect road cracks. They used mAP(mean average precision) as the performance metric to test the combinations of Object detecting paradigms and DCNNs. Among all the combinations, Faster RCNN paired with Inception V2 gave the best results with mAP at 53.06%. A two-stage detection model including a DCNN and a segmentation module was proposed by NHT Nguyen et al.^38^. The authors proved that the segmentation of cracks at the pixel level improves detection accuracy significantly. In a study presented by SE Park et al.^39^, cracks on concrete structure surfaces have been identified using DL and structured light technologies, which combine two laser sensors with vision.

The YOLO model was used to identify the cracks and the size of all cracks were calculated using the positions of the laser beams on the structural surface. Huyan et al.^40^, presented a model named CrackU-net which detects pavement cracks with a precision of 0.986. Kim et al.^41^, proposed a crack detection technique using shallow CNN architecture. They optimized the LeNet-5 model’s hyper-parameters to obtain maximum accuracy of 99.8% with fewer parameters. Even though some of these models performed pretty well in feature extraction and classification on various applications, their accuracy needs to be increased to detect concrete fractures. In this paper, we are evaluating the effectiveness of transfer-learned deep features for crack detection using raw and enhanced crack images, which shows a significant boost in terms of performance.

## Proposed methodology

**Figure 1:**
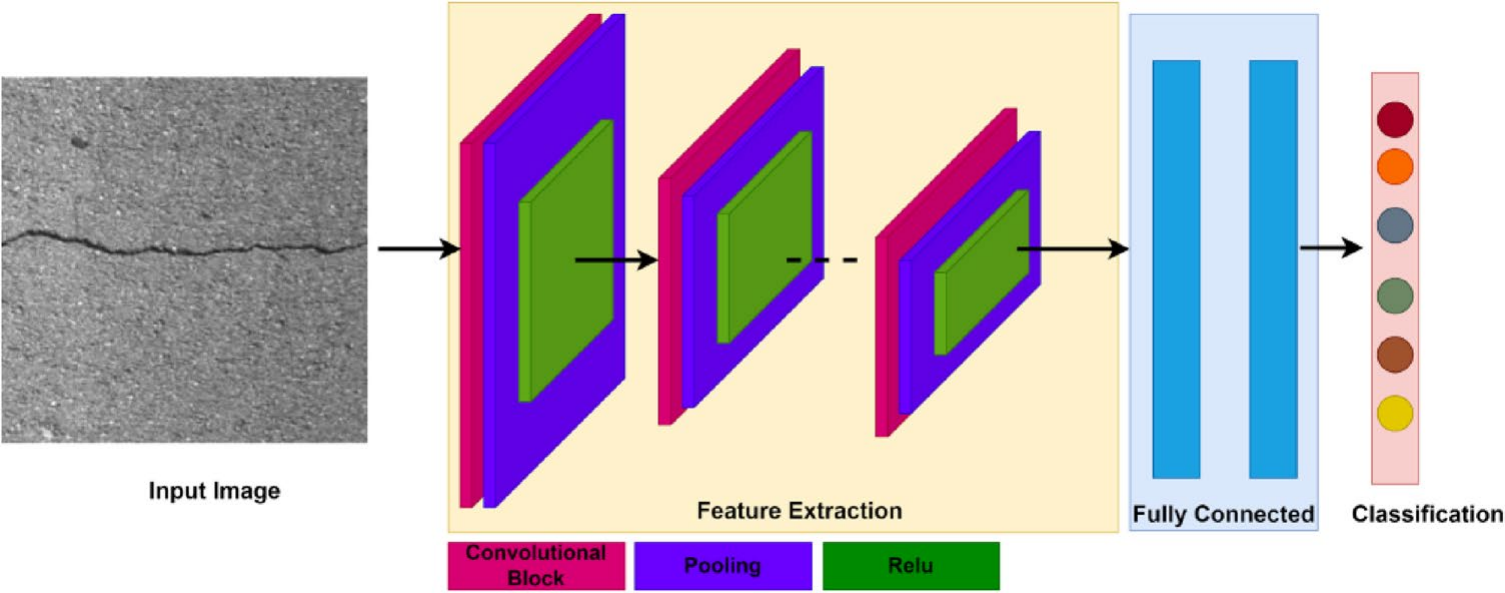
CNN Architecture.

This section introduces DCNNs and their application for crack detection in detail.DCNNs, which were first developed in the 1980s, is the most well-known, advanced, and popular DL algorithm^42^. Earlier the researchers were not drawn to DCNNs due to the availability of minimum computational resources, powerful processors, and huge storage devices. But when computers’ processing capacity for computing, database retrieval, and storage expanded, the idea gained popularity^43^. Later in^44^, CNN’s were successfully applied in classification problems and outperformed mostly in solving computer vision problems. Figure 1 depicts a typical CNN structure. The initial layers of DCNN extract basic image features such as edges, patterns, and textures. The middle layers extract object-level information like shape and color, whereas the deeper levels extract class-level features like the whole object. The feature extraction layer’s final output is passed into either a fully connected neural network^45^ for classification or a bounding box and pixel classification layer for segmentation.

CNN has emerged as the most widely used and successful DL architecture for various input data types including images, videos and texts, with several cutting-edge architectures reported in the literature. VGG16, VGG19^46^, Xception^47^, ResNet50, ResNet101, ResNet152^48^, InceptionV3^49^, InceptionResNetV2^50^, MobileNet^51^, MobileNetV2^52^, DenseNet121^53^, EfficientNetB0^54^ are some of the well-known and leading-edge DCNN architectures for classification. DCNN varieties for classification, segmentation, or localization can be used to detect cracks in the input image.

**Figure 2:**
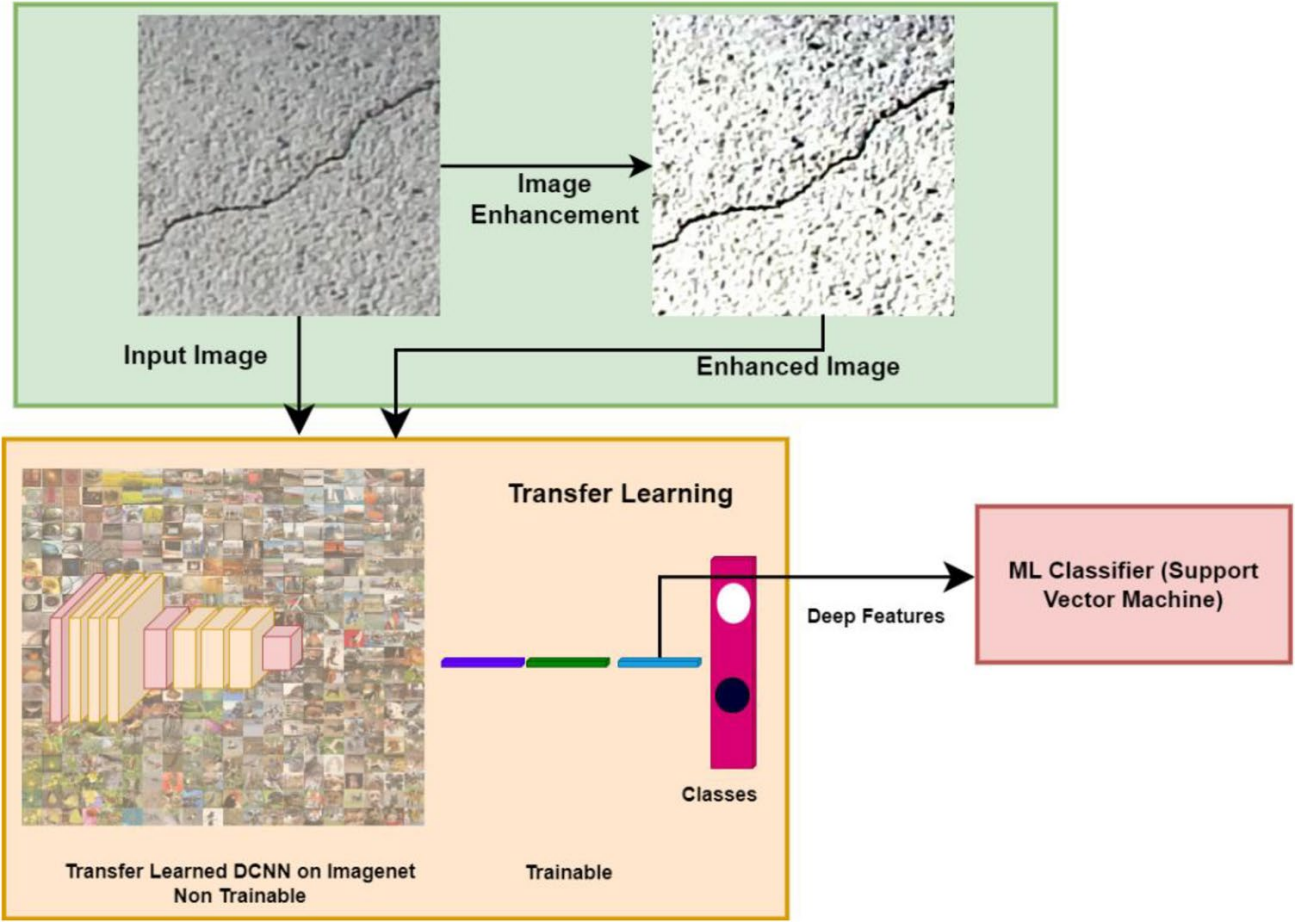
Proposed Transfer Learning Architecture for Crack Detection with Pre-trained CNN Models on ImageNet Weights.

This paper proposes transfer learning-based DL models for crack identification through classification. This work carried out three experiments: (1) Transfer Learning for Crack Detection Without Image Enhancement (2) Transfer Learning for Crack Detection with Image Enhancement (3) Crack detection using SVM on deep features. Figure 2 depicts the experiments carried out in the proposed model.

### Transfer learning for crack detection without image enhancement

A model created for one job is utilized as the basis for another task in transfer learning, a machine learning technique^55–57^. The use of pre-trained models as the foundation for computer vision and natural language processing tasks is a common strategy in DL research due to the massive computing time and resources required to develop neural network models^58^. The benefits of using a transfer learned model over an end-to-end neural network include significant time and computation savings. Recent research reveals that transfer-learned models outperform traditional neural networks and can work with smaller amounts of data. Generally, for computer vision applications, the features extracted by the first and middle layers of a neural network are similar for similar inputs. The latter layers that extract high-level features make the difference. The proposed model freezes the first and middle layers and makes the final layers trainable. We retain the weights from the old model trained on a comparatively large dataset and only train a few parameters.

**Figure 3:**
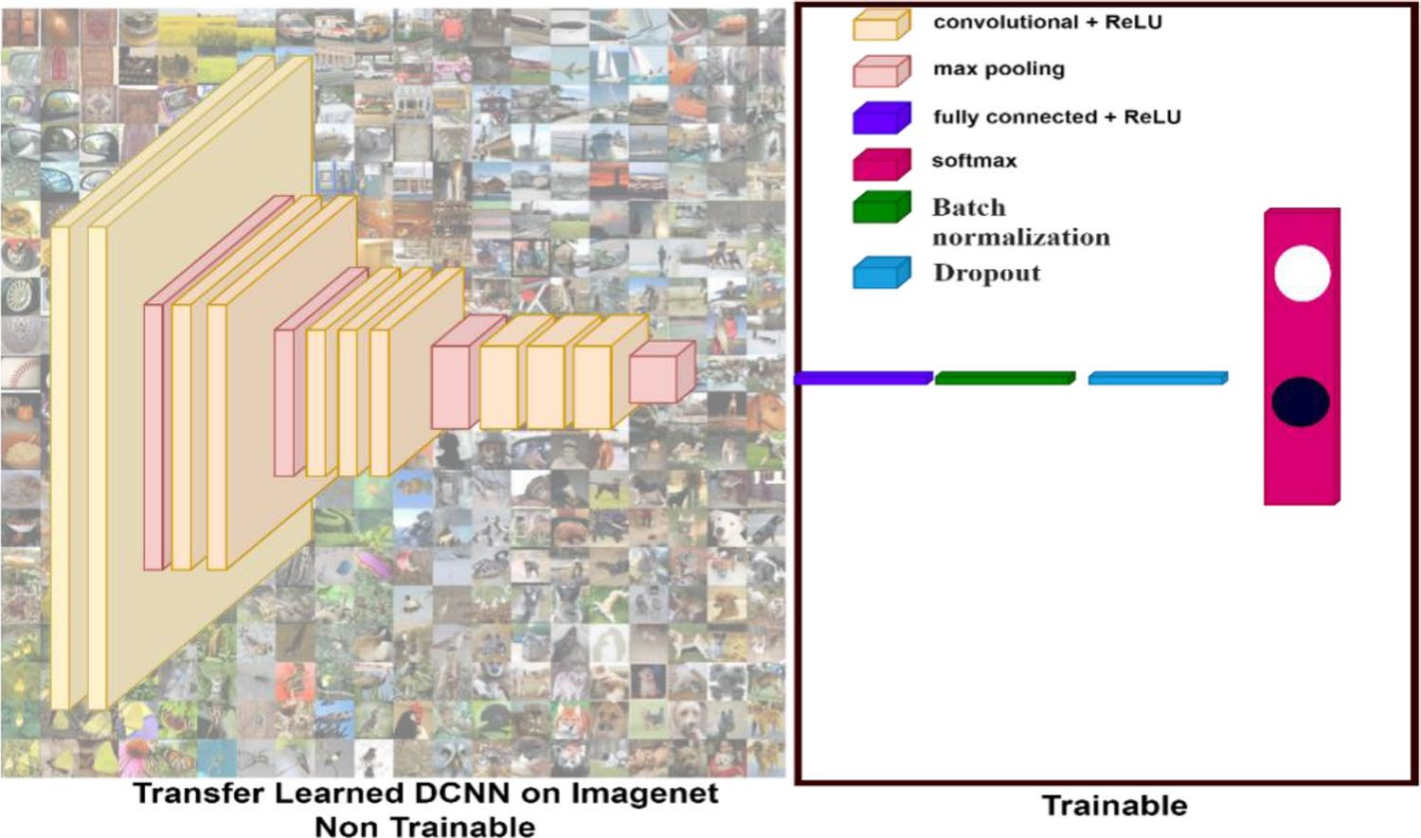
Transfer Learning Pipeline used in the proposed model.

Figure 3 illustrates the process of transfer learning applied to a Deep Convolutional Neural Network (DCNN) using pre-trained ImageNet weights. In this experiment, we adapted the DCNN model for crack detection by leveraging the weights learned from the ImageNet dataset.

To accomplish this, we first removed the final layers of the pre-trained models. These layers were then replaced with a new architecture consisting of several components: a flattened layer to convert the 2D feature maps into a 1D feature vector, a batch normalization layer to stabilize and accelerate the training process, a dropout layer to prevent overfitting by randomly setting a fraction of input units to zero during training, and a dense layer with two neurons, each using a sigmoid activation function to output the probability of the presence or absence of cracks.

Before training the model, the necessary datasets were collected. These datasets were then preprocessed by resizing the images to 224 × 224 pixels, a standard input size for many CNN architectures pre-trained on ImageNet. The resized dataset was subsequently split into three subsets: training, validation, and test sets. This division ensures that the model can be trained, validated, and tested on separate data to evaluate its performance accurately.After preparing the data, we loaded it into the pre-trained CNN model. As mentioned earlier, the model’s original final layers were replaced with a new set of custom layers. This new architecture was specifically designed to refine the pre-trained model’s capacity to detect cracks in images.

The transfer learning model was then trained, but with a specific focus on optimizing only a subset of parameters. Specifically, most parameters from the pre-trained layers were frozen, meaning they were not updated during training. Only the parameters from the newly added custom layers were fine-tuned. This approach allows the model to retain the general features learned from the ImageNet dataset while adapting its final layers to the specific task of crack detection with a smaller amount of data and computational resources.

### Transfer learning for crack detection with image enhancement

Two image enhancement methods: Local Binary Pattern and contrast enhancement were employed to pre-process the input image to train the DCNN models. Image enhancement modules were introduced with the assumption that when trained on enhanced input images, Convnets would easily converge, lowering computational costs and improving accuracy. The assumption was supported further by various benchmark evaluation metrics, as shown in the following sections. The selection of image enhancement algorithms was done based on the literature as proposed by Wang et al.^59^, and Chen et al.^60^.

### Contrast enhancement

Contrast enhancement in the image makes dark areas darker and light areas lighter, making cracks appear darker than other surfaces. This creates a significant difference between the dark and light areas, which will aid in subsequent classification^59^.

**Figure 4:**
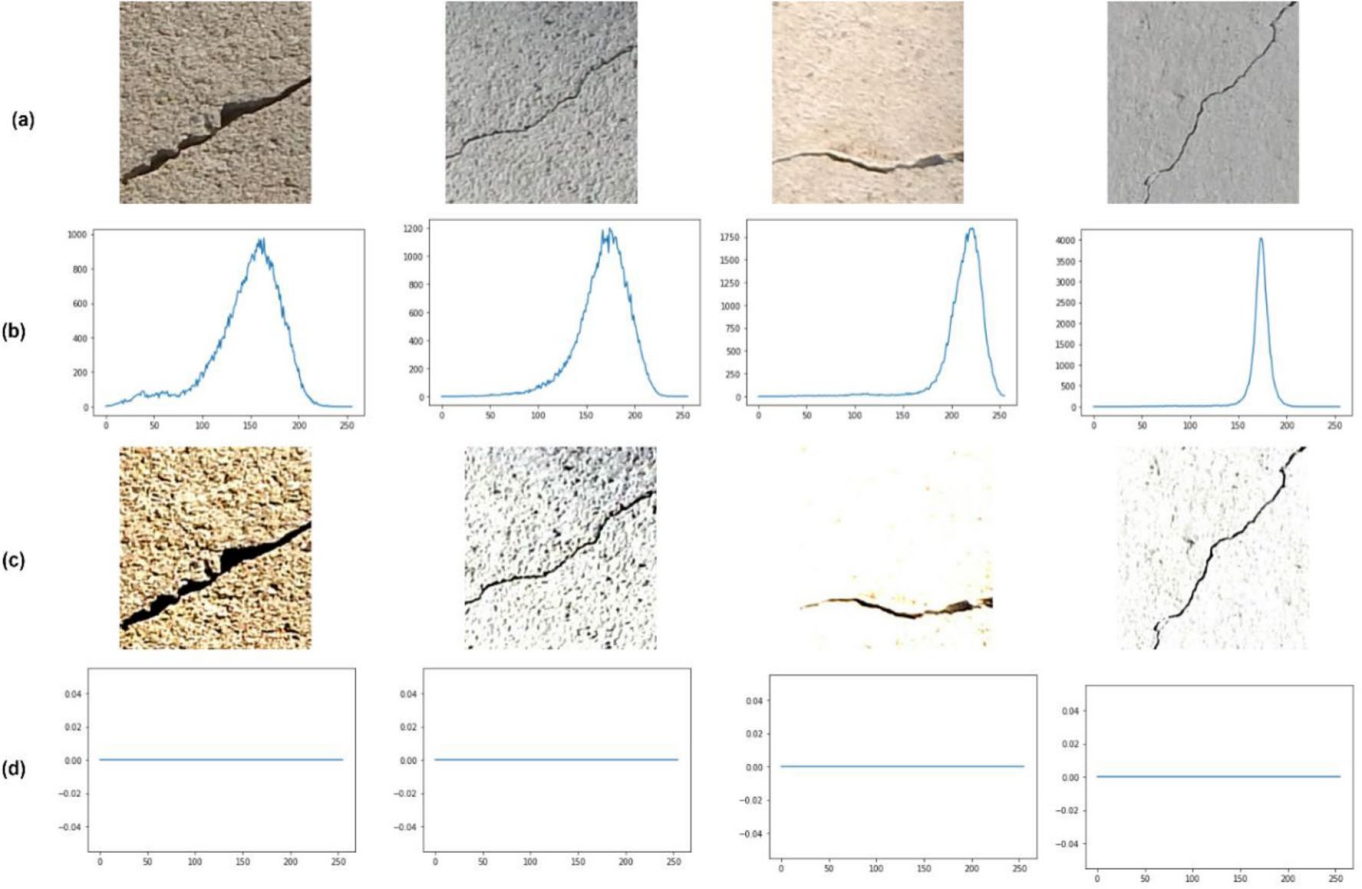
Contrast enhancement on the crack images. (**a**) Original image (**b**) Histograms of original images (**c**) Contrast-enhanced image (**d**) Histograms of Contrast-enhanced image.

Algorithm 1 details the steps followed for contrast enhancement and Fig. 4 shows the results of contrast enhancement on crack images selected randomly from the dataset. Figure plots the histograms corresponding to the original images and the contrast-enhanced images. From the figure, it is evident that the histograms of original crack images are not uniform (skewed towards the right) whereas that of enhanced images are uniform.

### Local binary pattern (LBP)

LBP is a primitive texture operator that labels pixels in an image by thresholding each pixel’s vicinity based on the current pixel^61^. It is considered an efficient descriptor due to its resistance to changes in illumination, computational simplicity, and reliability in image classification. The LBP Algorithm divides the image into smaller cells and uses the intensity of the center pixel as a threshold for the remaining pixels in the cell. When neighboring pixels are greater than the threshold value, they are thresholded to 1; otherwise, they are thresholded to 0. The binary number is generated by circularly visiting the matrix. As a result, the formed binary number is converted to a decimal and used to update the value of the center pixel.

**Table:** 

Algorithm 1: Contrast Enhancement Algorithm	
1	Take an input image, brightness value and contrast value
2	Check if brightness is equal to 0, if yes go to step 3 else go to step 5
3	If the brightness value is greater than 0 then assign brightness value to shadow and highlight to 255, else assign shadow to 0 and highlight to 255 + brightness value. Calculate the *alpha b* and *gamma b* values using the highlight and shadow values using the below formulas *alpha_b* = *(highlight – shadow)/255* *gamma b* = *shadow*
4	Using *input img*, *alpha b* and *gamma b* as inputs blend the images using add weighted function
5	Create an extra copy of the image
6	Check if the contrast value is not 0, if yes assign the variables *alpha c* and *gamma c* using the below formulas *alpha c* = 131*(contrast + 127) / (127*(131 – contrast)) gamma_c = 127*(1 – alpha_c)
7	Using *input img*, *alpha c* and *gamma c* as inputs blend the images using add weighted function

**Table:** 

Algorithm 2: Local Binary Pattern (LBP)	
1	Take a center pixel from the given image
2	Compare the value of the central pixel to the values of the 8 pixels in the vicinity
3	If the neighboring pixel’s value is greater than that of the center pixel then that particular pixel is assigned the value 1, else it is assigned 0
4	Replace the center pixel’s value using the neighboring 8 pixels as shown below: C = Σ (p_i_)*(2^i^), where 0 ≤ i ≤ 7
5	For each pixel in the provided image, repeat the preceding instructions

**Figure 5:**
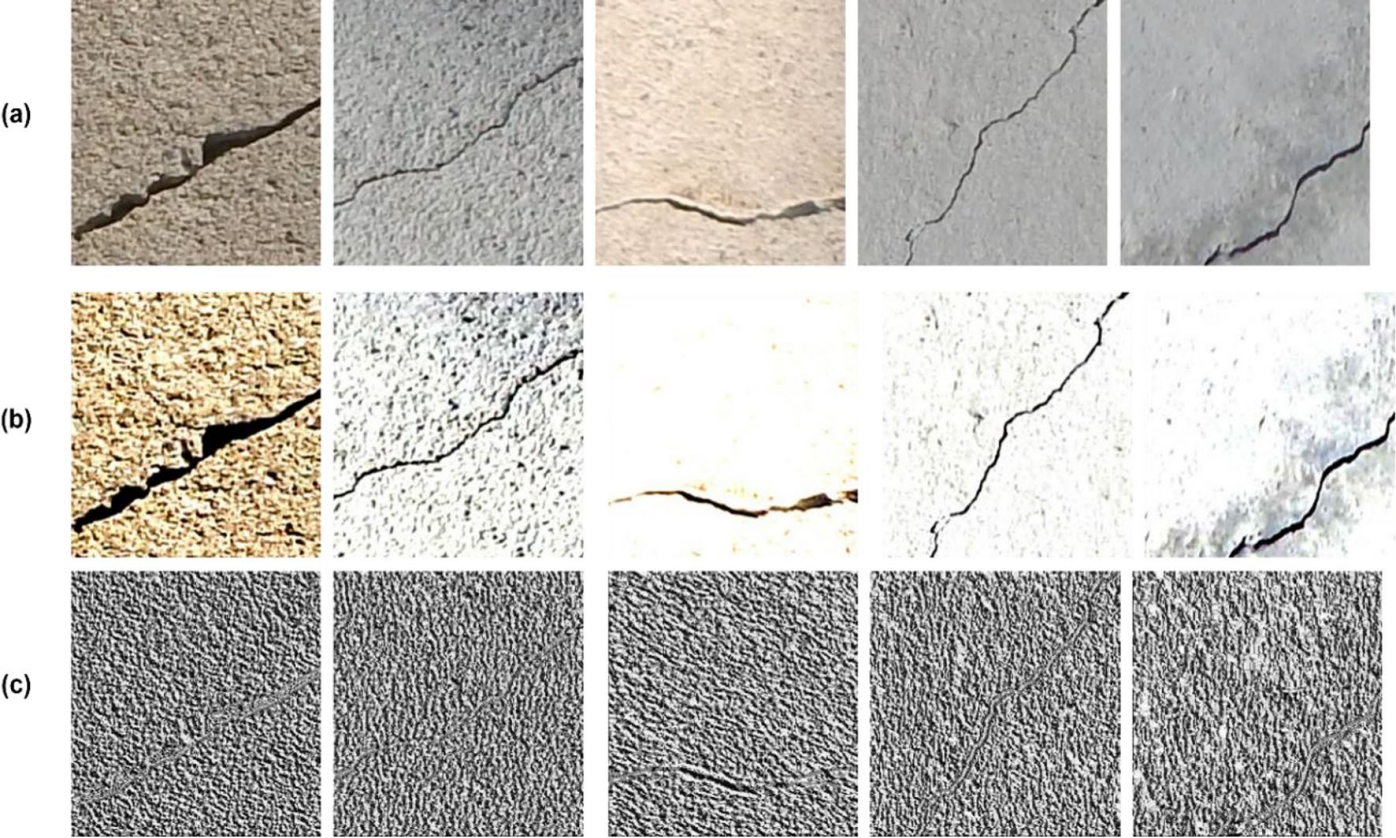
Image enhancement results on random images from SDNET^1^. (**a**) Original image (**b**) Contrast-enhanced images (**c**) LBP-processed Images.

The LBP feature descriptor is mathematically represented as follows:

$$LBP_{P,R} = \sum\limits_{p = 0}^{P - 1} {s\left( {c_{p} - n_{p} } \right)} 2^{p} s\left( x \right) = \left. {\left\{ {1,x \ge 00,x < 0} \right.} \right|$$

where *R* is the radius and *P* denotes the pixels adjacent to it. *c*_*p*_ is the center pixel’s grayscale value, and *n*_*p*_ is the grayscale value of the neighboring pixel. The LBP algorithm is detailed in Algorithm 2. Figure 5 compares results obtained from the image enhancement module (Contrast enhancement and LBP pre-processing) for random images from SDNET^8^. From Fig. 5, it is evident that the crack regions are more highly visible in the contrast-enhanced images than in the original and LBP pre-processed images.

Although the LBP operator attempted to get hold of the underlying texture of the input image, it was unable to highlight the cracked regions. The same is demonstrated by experimental results in terms of model accuracy on contrast-enhanced images and LBP pre-processed images as shown in Section “Transfer Learning for Crack Detection with Image Enhancement**”**.

### Crack detection using ML models based on deep features from DCNNs

**Figure 6:**
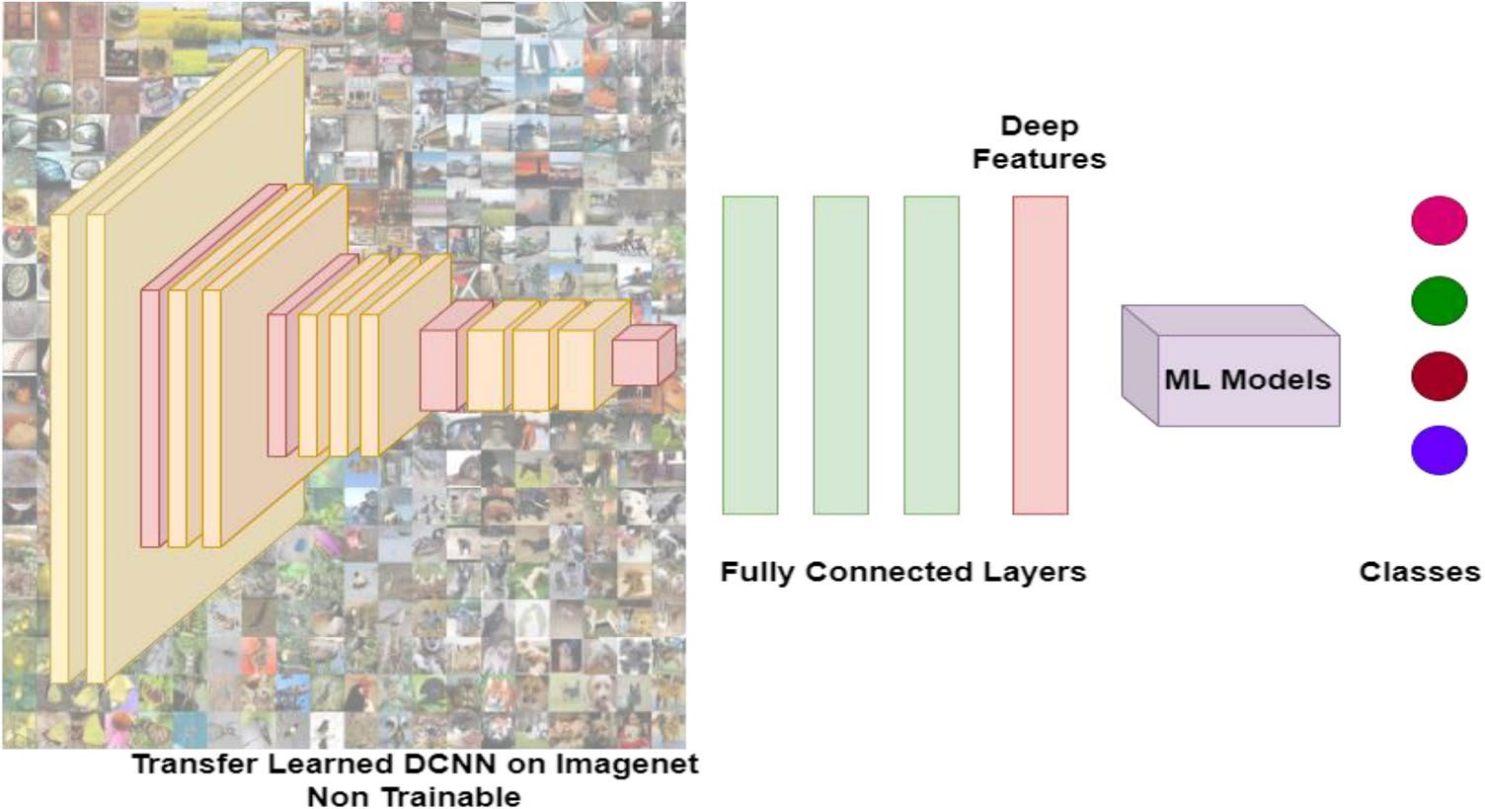
Crack Detection using ML models Based on Deep Features from DCNNs pipeline.

The effectiveness of deep features extracted from DCNN for classification is described in this section. The generic CNN architecture comprises a wide range of filters, pooling operators (Max pooling, Average Pooling), and nonlinear activations (ReLu, Sigmoid, Softmax). The filters are learned in either a supervised or unsupervised manner and extract relevant information from the input image. The pooling layers reduce the spatial dimension of the intermediate feature maps from convolution layers, and the activations introduce nonlinearity. Initial layers of DCNNs extract basic image features such as edges, textures, color etc. whereas the deeper layers extract complex class-specific features such as weights. This work proposes to use the weights learned by the deep layers of CNN as the feature representation for the input images, also known as Deep Features. Pre-trained CNN models including VGG16, VGG19, ResNet50, MobileNet, etc. were employed to extract the deep feature vectors to model the high-level representation of inputs. The extracted deep feature vectors are then fed into an ML algorithm like SVM for further classification as depicted in Fig. 6.

The choice of using deep feature representation for the classification using ML models is based on the assumption that ML models can produce accurate results when trained on good feature representation, and deep features extracted from the final layers of DCNNs can generate high-level representations, implying a symbiotic relationship.

## Dataset

This section details the dataset used for the experiment. Three publicly available datasets were used for the study SDNET2018^9^, Concrete Crack Images for Classification (CCIC)^10^ and Bridge Crack Dataset (BCD)^11^. We have formatted the dataset to have equal data points in all classes. However, class imbalance [69] can result in different results.

### SDNET dataset

The SDNET dataset includes 56,092 images of cracked and non-cracked bridges, pavement, and wall surfaces. Images of bridge decks were obtained from the Systems, Materials, and Structural Health (SMASH) Laboratory at Utah State University, which houses a variety of full-scale bridge deck sections. Images of walls and pavements were taken on the premises of the Utah State University campus. All of the images are 256 × 256 pixels in size and in.jpg format. Table 1summarizes the number of crack and non-crack images in each subclass of the SDNET dataset (bridge decks, walls, pavement).

**Table 1:** SDNET dataset.

	Crack	Non–Crack	Total
Bridge	2025	11,595	13,620
Walls	3851	14,287	18,138
Pavements	2608	21,726	24,334
Total	8484	47,608	56,092

### CCIC dataset

The CCIC dataset includes images of concrete cracks and non-cracks. It includes more than 40,000 pictures gathered from different METU campus buildings. This dataset is balanced with only one type of surface concrete. It has 20,000 images in each class, crack and non-crack respectively. The images are of size 227 × 227.

### Bridge crack dataset (BCD)

Over 6070 images of cracked and uncracked bridge surfaces are included in the Bridge Crack Dataset (BCD). The crack images were captured using the Phantom 4 Pro’s 1024 1024 CMOS surface array camera. The images were later reduced to 224 × 224 dimensions to create the dataset. This dataset contains 4056 cracked images and 14 non-cracked images. The details of the count of crack and non-crack images of the 3 datasets are provided in Table 2.

**Table 2:** Crack and Non-Crack image distribution in SDNET2018, CCIC and BCD datasets.

	SDNET2018	CCIC	BCD
Crack	8484	20,000	4056
Non-crack	47,608	20,000	2014
Total	56,092	40,000	6070

**Figure 7:**
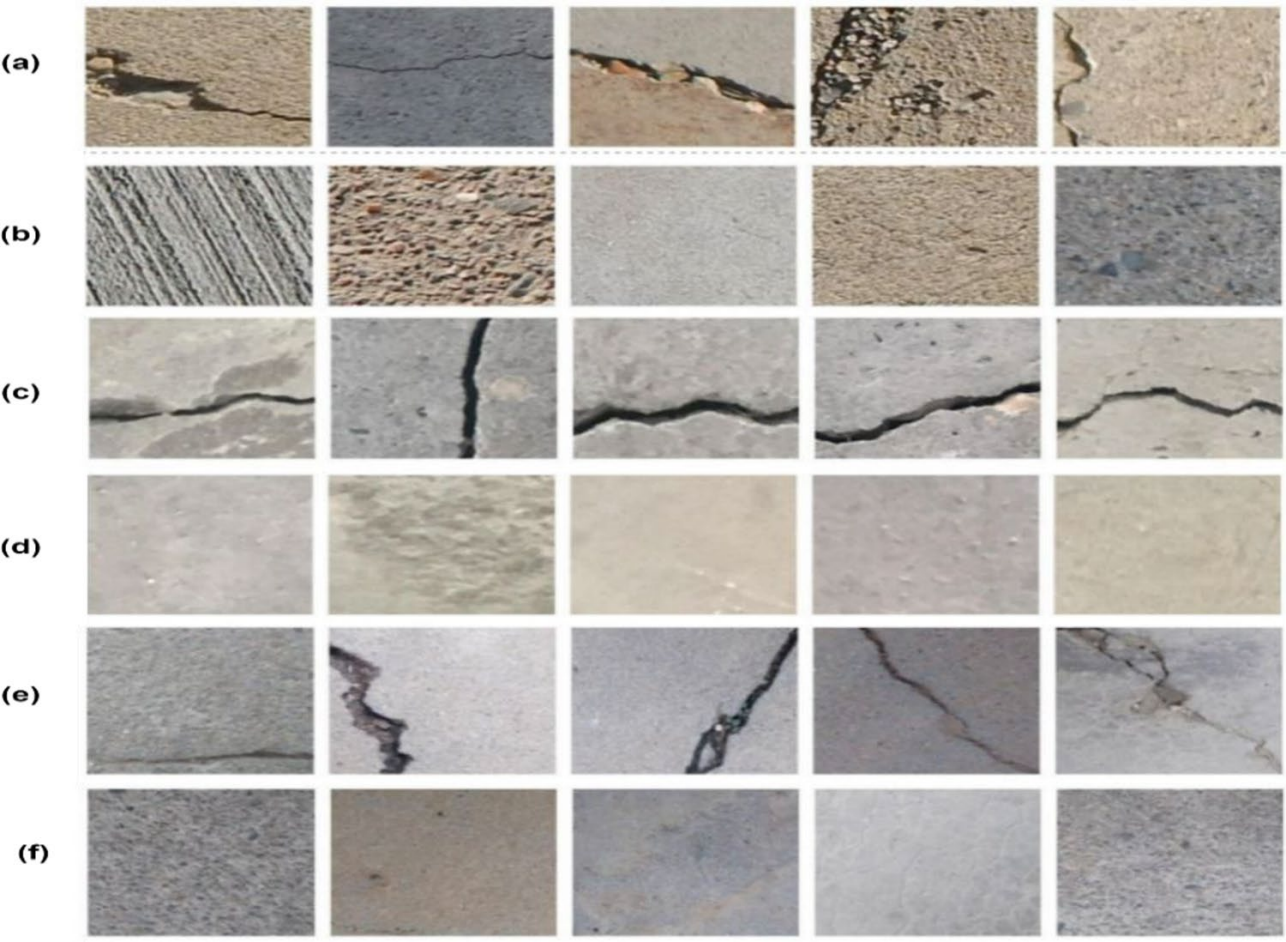
Sample crack and non-crack images from the three datasets. (**a**) Crack images from SDNET (**b**) non-crack images from SDNET (**c**) Crack images from CCIC (**d**) non-crack images from CCIC (**e**) Crack images from BCD (**f**) non-crack images from BCD.

Since all these datasets are quite large, we conducted the experiments with a smaller number of images from each of them. Table 3summarizes the train and validation split of the images used for experiments for the three datasets and Fig. 7 shows sample images from the three datasets.

**Table 3:** Train-Validation split of SDNET2018, CCIC and BCD datasets.

		SDNET2018	CCIC	BCD
Train	crack	1000	750	1000
	non-crack	1000	750	1000
Validation	crack	500	250	500
	non-crack	500	250	500
Total		3000	2000	3000

## Experimental result and analysis

This section details the obtained results and their analysis using benchmark evaluation metrics. The performance of classification models for crack detection with 12 image classification models (VGG16, VGG19, Xception, ResNet50, ResNet101, ResNet152, InceptionV3, InceptionResNetV2, MobileNet, MobileNetV2, DenseNet121 and EfficientNetB0) on 3 different datasets (SDNET, CCIC, BCD) were experimented.

### Hardware and software specifications

The models were implemented on Google Colaboratory and Jupyter notebook with the Machine Learning and Deep Learning packages. The hardware specifications used for the experiments are listed in Table 4.

**Table 4:** Hardware and Software Specifications.

Hardware	Specification
Device	Laptop
RAM	8 GB
HDD	1 TB
SSD	128 GB
OS	Windows 10 Home
Graphics	4 GB Graphics—Nvidia GTX 1650

### Performance measures

Accuracy, sensitivity, specificity, precision, recall, F1-score, and training duration were used to assess each model’s performance. The confusion matrix, which is used to determine the model’s overall performance and is displayed in Table 5, is utilized to calculate the performance metrics shown below.

**Table 5:** Confusion matrix.

Actual class			
Predicted Class		Positive (1)	Negative (0)
	Positive (0)	True Positive (TP)	False Positive (FP)
	Negative (0)	False Negative (FN)	True Negative (TN)

### Accuracy

Number of predictions made correctly by the model concerning the total predictions made.

$$Accuracy = \, (TP + TN)/(TP + FP + TN + FN)$$

### Precision

Measure of quality of how good the model is at predicting a particular category.

$$Precision = \, {\mathbf{TP}}/\left( {{\mathbf{TP}} \, + \, {\mathbf{FP}}} \right)$$

### Recall or sensitivity

The proportion of Positive samples that were correctly identified as Positive to all of the Positive samples.

$$Recall = \, {\mathbf{P}}/\left( {{\mathbf{TP}} \, + \, {\mathbf{FN}}} \right)$$

### F1-Score

The harmonic mean of precision and recall are given by:

$${\mathbf{F1}} - {\mathbf{Score}} \, = \, \left( {{\mathbf{2}}*{\mathbf{precision}}*{\mathbf{recall}}} \right)/\left( {{\mathbf{precision}} \, + \, {\mathbf{recall}}} \right)$$

### Transfer learning on 3 datasets without image pre-processing

The study and findings of transfer learning without image enhancement are covered in this subsection. Using the ImageNet weights from the pre-trained model reduced the number of parameters that needed to be trained in this experiment. A flattened layer, batch normalization layer, dropout layer, and a dense layer with two neurons and a sigmoid as an activation function were added instead of the top layers of all the pre-trained models to achieve this. The transfer learning method’s fine-tuned hyper-parameters are tabulated in Table 6.

**Table 6:** Hyperparameters.

Hyperparameter	Values
Learning rate	0.001
Dropout rate	0.2
Number of epochs	50

**Figure 8:**
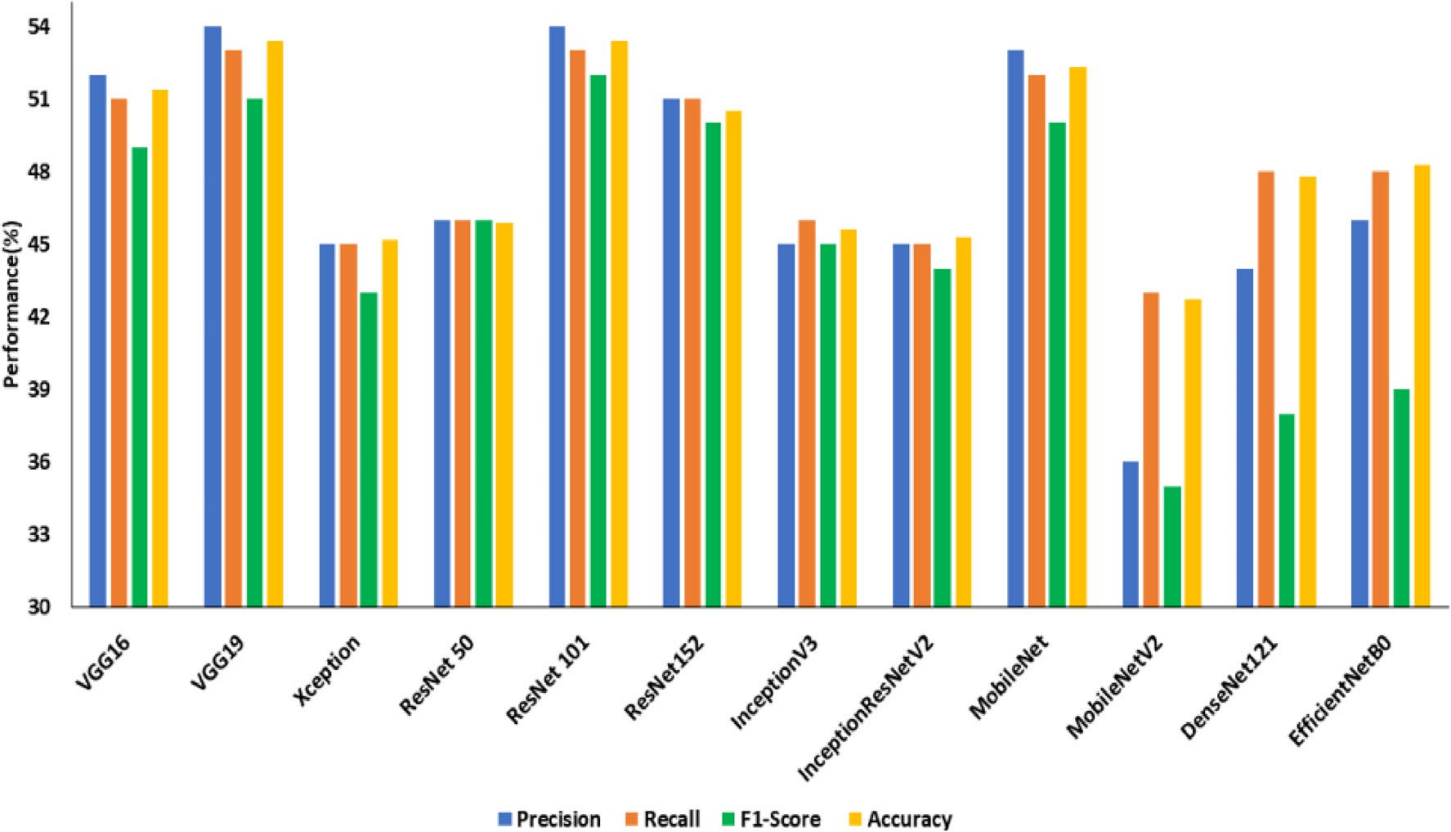
Performance comparison of transfer learned DCNNs on SDNET without image pre-processing.

**Figure 9:**
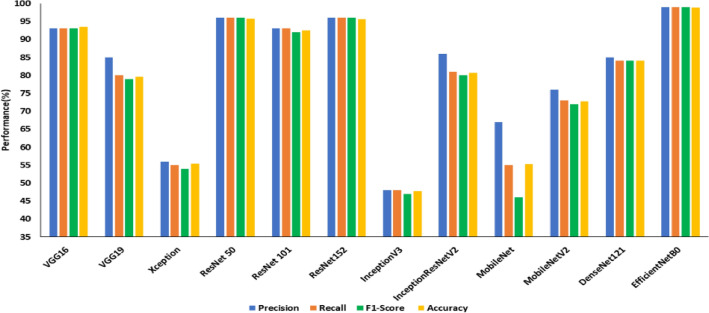
Performance of transfer learned DCNNS on CCIC without image pre-processing.

**Figure 10:**
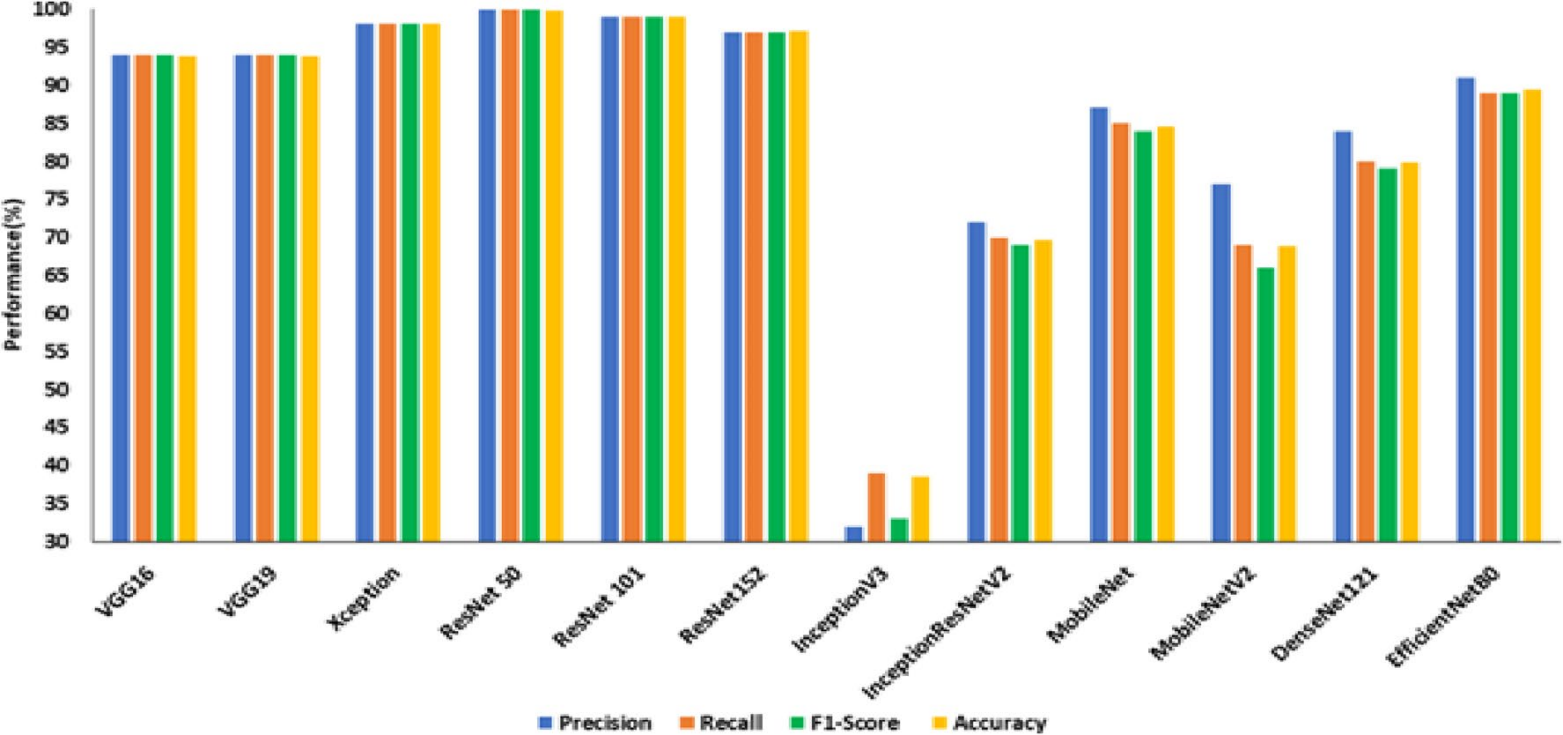
Performance of transfer learned DCNNS on BCD without image pre-processing.

Figures 8, 9 and 10 show the performance comparison (precision (%), recall (%), accuracy (%)) of state-of-the-art transfer learned DCNNs on SDNET, CCIC and BCD datasets, respectively.

From Fig. 8, it is observed that the ResNet101 is the best model on the SDNET dataset concerning test accuracy. In 34.45 min of training, the model achieved an accuracy of 53.40 percent. The model that performs the poorest concerning test accuracy is MobileNetV2, with a test accuracy of 42.7%. The best model on the BCD dataset from Fig. 9, EfficientNetB0, has a test accuracy of 98.8% and a training time of 30.15 min. InceptionV3 has the lowest test accuracy of 47.8% on the BCD dataset.The best model for the CCIC dataset is ResNet50 (refer to Fig. 9), which achieved a test accuracy of 99.8% after 25.18 min of training. InceptionV3 has the lowest test accuracy compared to other DCNNs on this dataset, with 38.6%.

Table 7 summarizes the precision, recall and F1 score obtained for the transfer of learned DCNNs on three publicly available datasets under study. From Table 7, it is evident that all the transfer-learned DCNNs perform poorly on the SDNET dataset compared to CCIC and BCD in terms of the three benchmark evaluation metrics under consideration. Based on this observation SDNET dataset was considered for the second experiment on transfer learned DCNNs using enhanced crack images.

**Table 7:** Summary of performance comparison of transfer learning for crack detection in terms of precision, recall and F1 score on three datasets.

Model	SDNET			CCIC			BCD		
	Precision	Recall	F1 Score	Precision	Recall	F1 Score	Precision	Recall	F1 Score
VGG16	52	51	0.49	94	94	0.94	93	93	0.93
VGG19	54	53	0.51	94	94	0.94	85	80	0.79
Xception	45	45	0.43	98	98	0.98	56	55	0.54
ResNet 50	46	46	0.46	100	100	0.10	96	96	0.96
ResNet 101	54	53	0.52	99	99	0.99	93	93	0.92
ResNet 152	51	51	0.50	97	97	0.97	96	96	0.96
InceptionV3	45	46	0.45	32	39	0.33	48	48	0.47
InceptionResNet V2	45	45	0.44	72	70	0.69	86	81	0.80
MobileNet	53	52	0.50	87	85	0.84	67	55	0.46
MobileNetV2	36	43	0.35	77	69	0.66	76	73	0.72
DenseNet121	44	48	0.38	84	80	0.79	85	84	0.84
EfficientNetB0	46	48	0.39	91	89	0.89	99	99	0.99

### Transfer learning for crack detection with image enhancement

**Figure 11:**
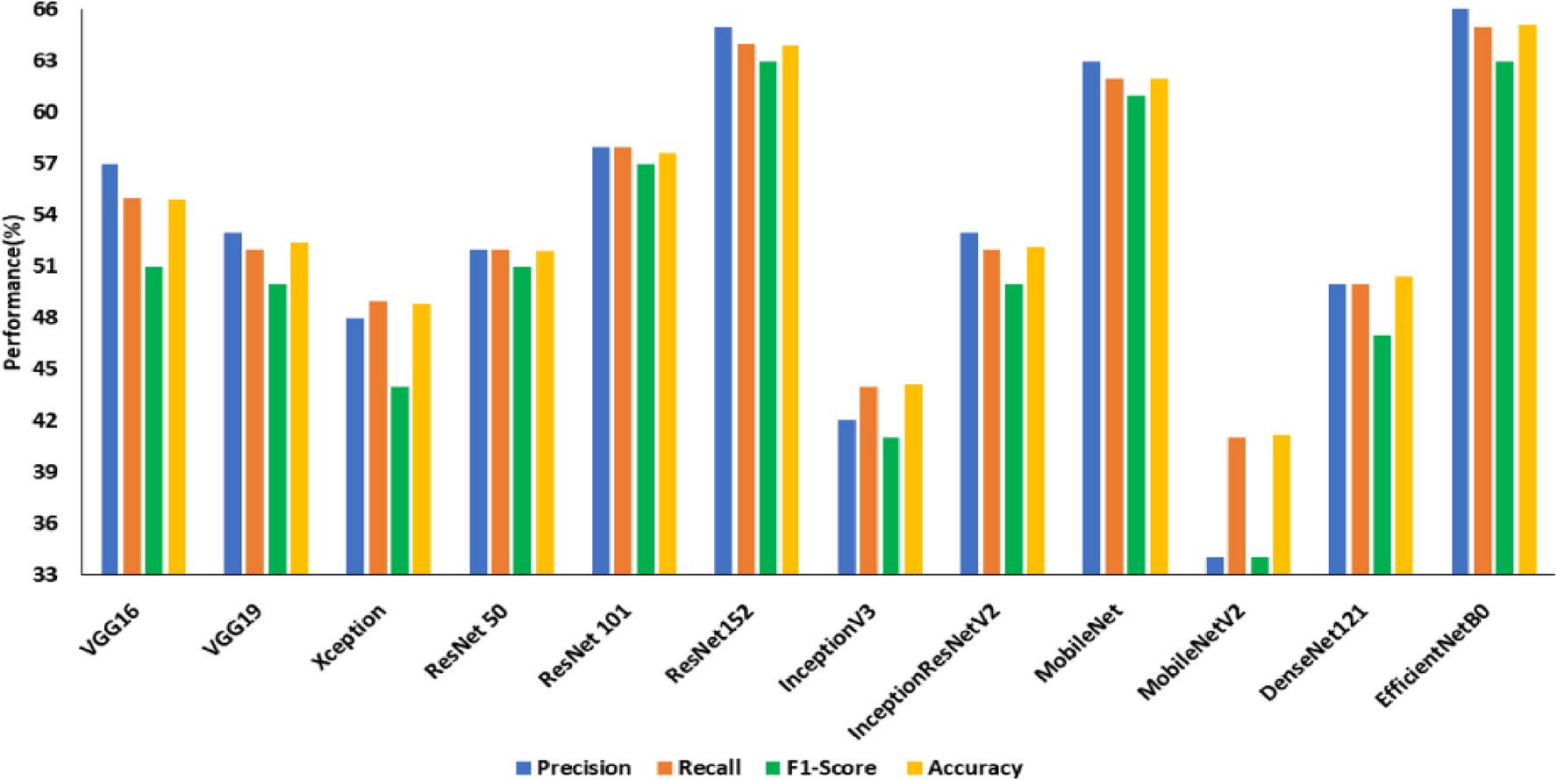
Performance of transfer learned DCNNS on SDNET with Contrast enhancement.

**Figure 12:**
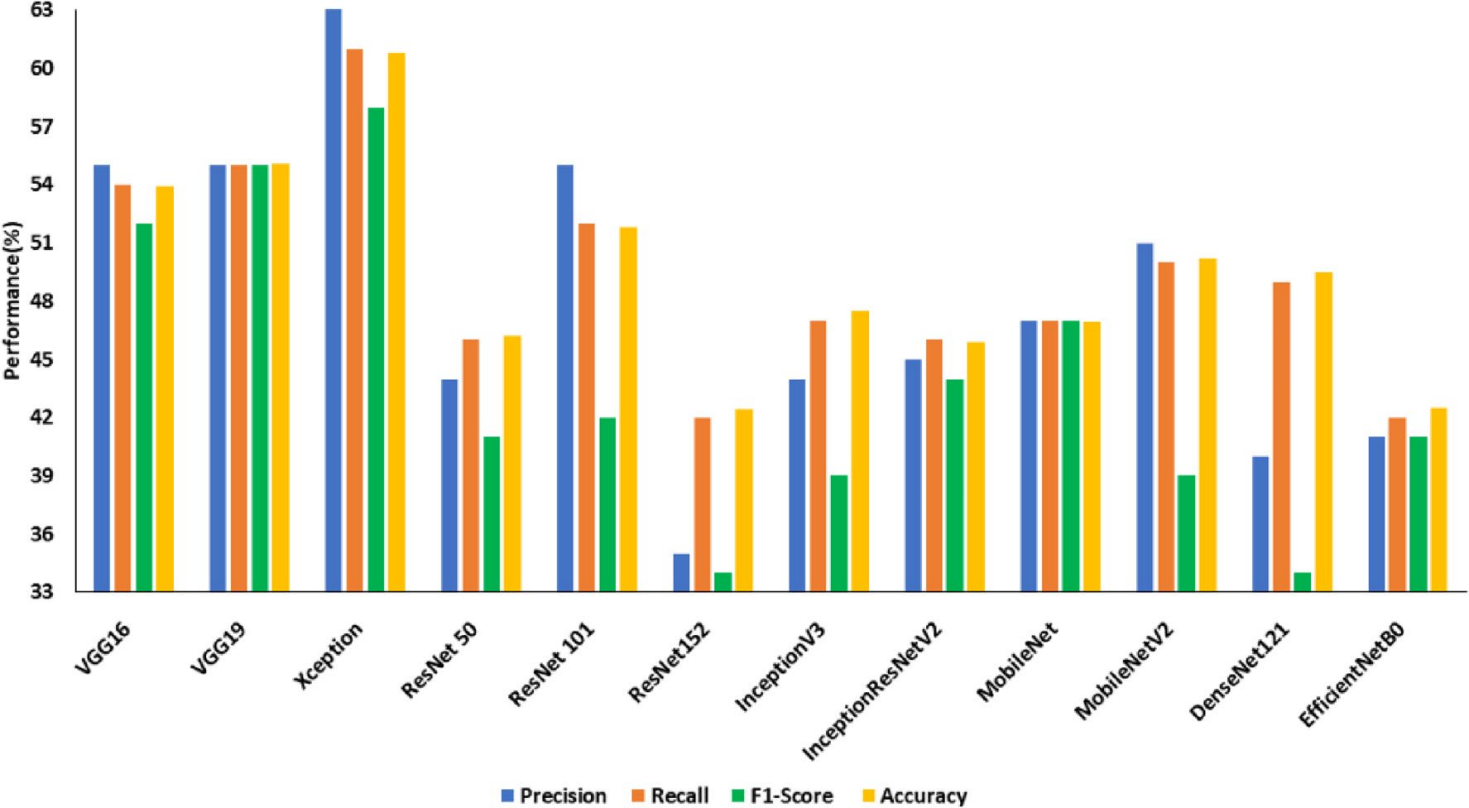
Performance of transfer learned DCNNS on SDNET with LBP pre-processing.

Images from the SDNET dataset were pre-processed using image enhancement algorithms, and the improved images were used to transfer and learn the DCNNs. Contrast enhancement and texture feature analysis using the LBP operator were employed to enhance the crack images. The transfer learnt models were then trained using the improved images. EfficientNetB0 achieved the highest test accuracy of 65.10% on contrast-enhanced images (an improvement of 16.8%), whereas a test accuracy of 41.20% was achieved by MobileNetV2. The model that fared the best among those trained using LBP-added images was Xception, with a test accuracy of 60.80% (an improvement of 15.6%), whereas ResNet152 underperformed with a test accuracy of 42.40%. Figure 11 and Fig. 12 compare the performance of transfer learned DCNNs on contrast-enhanced images and LBP pre-processed images respectively.

Table 8 compares the improvement without and with image enhancement on SDNET images. Highlighted improvements include those in recall, precision, and F1 score. It is evident from the table that contrast enhancement improved the performance of most of the deep CNN architecture under consideration for crack detection since the enhanced images were able to highlight the cracked regions better than that of normal images.

**Table 8:** Comparison of transfer learned models with/without image enhancement on SDNET dataset in terms of precision, recall and F1 score.

Model	Precision without enhancement (%)	Precision with enhancement (%)	Difference in Precision (%)	Recall without enhancement (%)	Recall with enhancement (%)	Difference in Recall (%)	F1 Score without enhancement (%)	F1 Score with enhancement (%)	Difference in F1 Score (%)
VGG16	52	57	5( +)	51	55	4( +)	0.49	0.51	0.02( +)
VGG19	54	53	1(−)	53	52	1(−)	0.51	0.50	0.01(−)
Xception	45	48	3( +)	45	49	4( +)	0.43	0.44	0.01( +)
ResNet 50	46	52	6( +)	46	52	6( +)	0.46	0.51	0.05( +)
ResNet 101	54	58	4( +)	53	58	5( +)	0.52	0.57	0.05( +)
ResNet 152	51	65	14( +)	51	64	13( +)	0.50	0.63	0.13( +)
InceptionV3	45	42	3(−)	46	44	2(−)	0.45	0.41	0.04(−)
InceptionResNet V2	45	53	8( +)	45	52	7( +)	0.44	0.50	0.06( +)
MobileNet	53	63	10( +)	52	62	10( +)	0.50	0.61	0.11( +)
MobileNetV2	36	34	2(−)	43	41	2(−)	0.35	0.34	0.01(−)
DenseNet121	44	57	7( +)	48	50	2( +)	0.38	0.47	0.09( +)
EfficientNetB0	46	70	24( +)	48	65	17( +)	0.39	0.63	0.24( +)

### Experiment 3: Crack detection using ML models based on deep features from DCNNs

**Figure 13:**
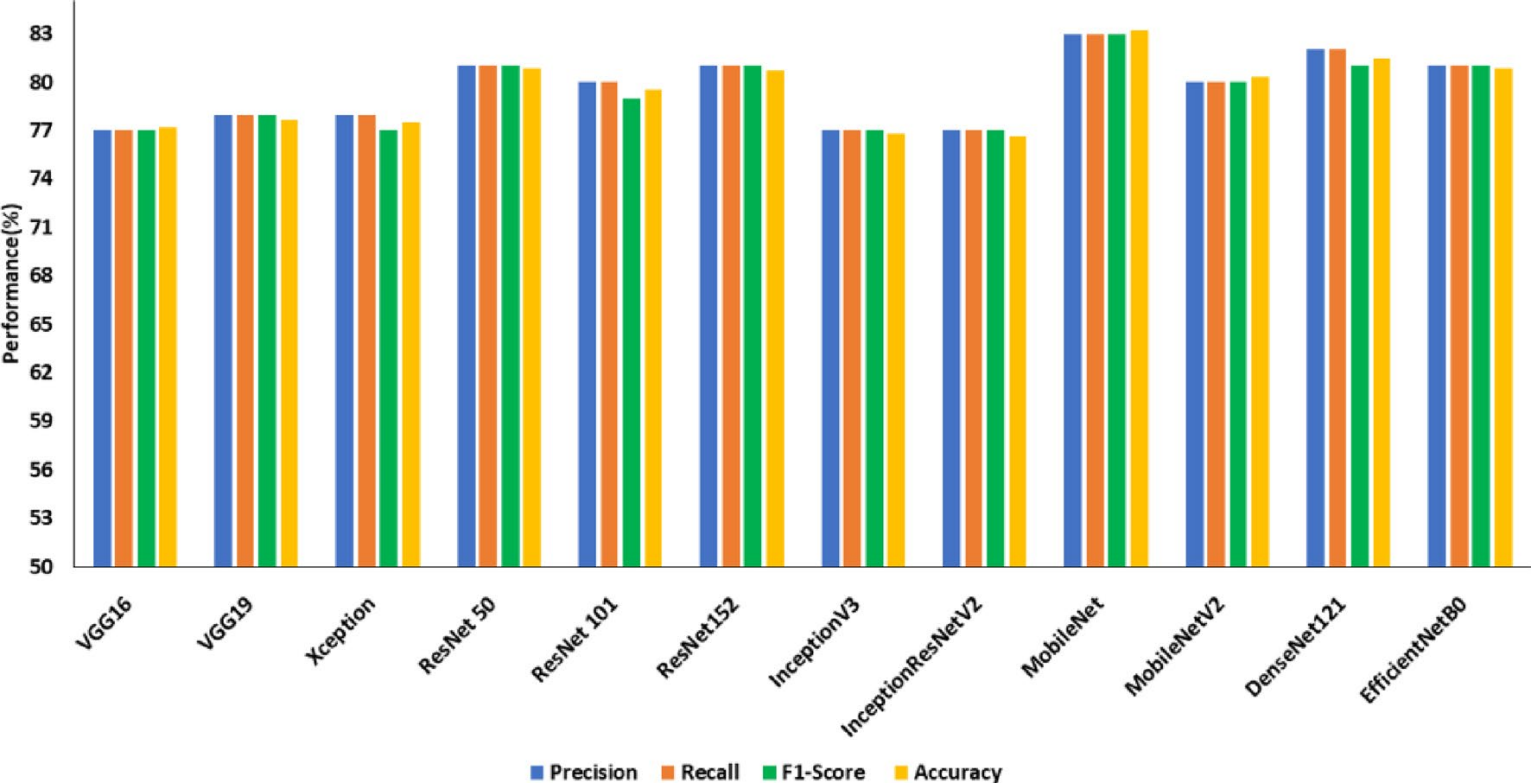
Performance of SDNET with deep features.

**Figure 14:**
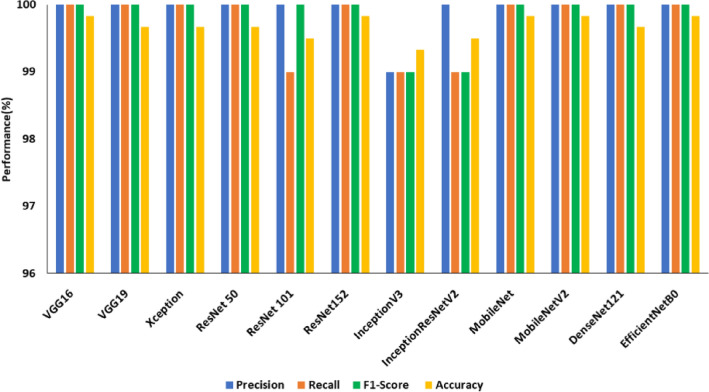
Performance of CCIC with deep features.

Deep features extracted from the final fully connected layers of DCNNs and Support Vector Machine (SVM) are employed in this subsection to categorize the images into crack and non-crack classes. SVM is the most appropriate model to handle datasets with fewer samples of high-dimensional features because the deep features extracted from the fully connected layers of DCNNs will be high-dimension in nature^62^. Deep features and SVM increased the overall accuracy of the models for classification as tabulated in Table 9. From Fig. 13, it is understood that the MobileNet produced an accuracy of 83.16% (best model) on the SDNET dataset with deep features and SVM, while VGG16 has an accuracy of 77.16%. All the 12 deep CNN models were able to achieve an accuracy greater than 99% on the CCIC dataset which is shown in Fig. 14. The models VGG16, ResNet152, MobileNet, MobileNetV2, and EfficientNetB0 continue to be the most accurate in this category with a 99.83% accuracy. Among the aforementioned top 5 DCNNs in terms of accuracy, MobileNetV2 has the fewest training parameters (2,223,872). From the observations, it can be inferred that MobileNetV2 demonstrated the optimum trade-off between accuracy and trainable parameters on the CCIC dataset.

**Table 9:** Comparison of ML models based on deep features in terms of accuracy.

Model	SDNET			CCIC		BCD	
	Accuracy without image enhancement	Accuracy with image enhancement	Accuracy of ML models with deep features	Accuracy without image enhancement	Accuracy of ML models with deep features	Accuracy without image enhancement	Accuracy of ML models with deep features
VGG16	51.4	54.9	77.16	93.8	99.83	93.4	99.83
VGG19	53.4	52.4	77.66	93.8	99.67	79.6	99.67
Xception	45.2	48.8	77.5	98	99.67	55.4	99.67
ResNet 50	45.9	51.9	80.83	99.8	99.67	95.8	99.67
ResNet 101	53.4	57.6	79.5	99	99.5	92.5	99.5
ResNet 152	50.5	63.9	80.66	97.2	99.83	95.6	99.83
InceptionV3	45.6	44.1	76.83	38.6	99.83	47.8	99.83
InceptionResNet V2	45.3	52.1	76.66	69.6	99.5	80.7	99.5
MobileNet	52.3	62	83.16	84.6	99.83	55.3	99.83
MobileNetV2	42.7	41.2	80.33	68.8	99.83	72.7	99.83
DenseNet121	47.8	50.4	81.5	79.8	99.67	84	99.67
EfficientNetB0	48.3	65.1	80.83	89.4	99.83	98.8	99.83

**Figure 15:**
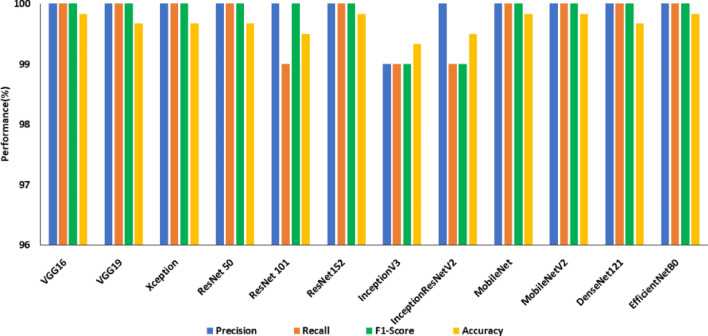
Performance of BCD with deep features.

From Fig. 15, it is observed that the best models on the BCD dataset are ResNet101 and EfficientNetB0, both of which have an accuracy of 99.83%. EfficientNetB0 is preferred over ResNet101 as it has a smaller number of trainable parameters—nearly ten times fewer.

Table 9 compares the improved ML models based on deep features for all three datasets. MobileNet was the model that performed the best among the SDNET models, which witnessed an increase in accuracy of between 20 to 30%. For the CCIC dataset accuracy enhancement is 10% and for the BCD dataset 11%.

### Overall inference

The proposed study employed 12 pre-trained CNN models to get the best performance for identifying crack and non-crack surfaces. InceptionV3, InceptionResNetV2, MobileNet, MobileNetV2, DenseNet121, and EfficientNetB0 deep models were used to assess the performance of deep feature extraction and transfer learning. It can be shown from the 3 datasets (SDNET, CCIC, and BCD) that the models did exceptionally well on the CCIC and BCD datasets. This is because each of these datasets has a consistent dataset with just one type of surface. The SDNET dataset, on the other hand, has many cracks and non-crack images of various surfaces. This makes it challenging for the models to achieve the necessary accuracy on the SDNET dataset. Transfer learning models performed well on the SDNET dataset, with ResNet101 outperforming the others. ResNet50 and EfficientNetB0 were the best-performing models on the CCIC and BCD datasets, respectively. Even though some models did well on the CCIC and BCD datasets, their accuracy could yet be improved. The findings of the following experiment, in which deep features were extracted and SVM was used to classify data, were better than those of the prior one. MobileNet was the model that performed the best among the SDNET models, which witnessed an increase in accuracy of between 20 to 30%. On the CCIC and BCD datasets, each model’s accuracy was close to 99%. The model’s accuracy significantly improves when extracted deep features are fed to the SVM classifier. The accuracy of models on the SDNET dataset could yet be increased. In other words, performance measures were assessed after all of these models underwent training using images that had previously experienced some processing. While the texture operator LBP did not significantly affect model accuracy, increasing contrast proved to be a helpful pre-processing strategy that led to greater accuracy. This experiment outperformed the prior transfer learning models, but not the accuracy attained by deep features fed into the SVM classifier. From Table 10 and Table 11 it is inferred that, out of the three experiments, classifying images as crack or non-crack using deep features provided to the SVM classifier was successful and produced superior accuracies across all datasets (SDNET: MobileNet; CCIC: MobileNetV2; BCD: EfficientNetB0).

**Table 10:** Best model for all three datasets over different experiments.

	Without image processing	LBP image processing	Image contrast enhancement	Deep features + ML
SDNET	ResNet101	Xception	EfficientNetB0	MobileNet
CCIC	ResNet50	ResNet50	EfficientNetB0	MobileNetV2
BCD	EfficientNetB0	EfficientNetB0	ResNet50	EfficientNetB0

**Table 11:** Maximum accuracy for all three datasets over different experiments.

Accuracy (%)	Without image processing	LBP image processing	Image contrast enhancement	Deep features + ML
SDNET	53.4	60.8	65.1	83.16
CCIC	99.8	88.2	99.2	99.83
BCD	98.8	88.3	95.2	99.83

## Conclusion and future scope

The proposed study compared the effectiveness of Deep Convolutional Neural Networks as a classifier and as a feature extractor for crack detection.

- The performance of 12 different transfer-learned DCNN models for crack detection was evaluated and analyzed on three publicly available datasets: SDNET, CCIC and BCD. The effectiveness of image enhancement and deep features extracted from the final fully connected layers of CNN models for classification was also analyzed in terms of benchmark evaluation metrics.
- ResNet101(Accuracy: 53.40%), EfficientNetB0 (Accuracy:98.8%) and ResNet50(Accuracy:99.8%) produced best accuracy with normal images from SDNET, BCD and CCIC dataset respectively. Since the effectiveness of transfer learned deep models were minimal on the SDNET images, two image enhancement methods (contrast enhancement and Local Binary Pattern) were employed on the images.
- The experimental results show that the enhanced images improved the accuracy of transfer-learned crack detection models significantly.
- The effectiveness of Deep features extracted from the final fully connected layers of DCNNs was analyzed in terms of classification accuracy. The extracted deep feature was fed into SVM for classification and the analysis in terms of accuracy, precision, recall, and F1-score revealed that the integration of deep features with SVM improved the detection accuracy across all the DCNN-dataset combinations.
- Among the SDNET models, MobileNet was the finest model, with an improvement in accuracy of between 20 and 30%. Each model’s accuracy on the CCIC and BCD datasets was close to 99% for MobileNetV2 and EfficientNetB0 respectively.

The main takeaway is that we can enhance the efficiency, accuracy and decision-making processes in civil engineering applications using these models. By using ML/DL models, the task of structural health monitoring becomes so easy and efficient. It identifies potential structural issues in early stages, contributing to faster maintenance and better safety. A custom ensemble model by combining the best DCNNs for crack detection could be considered as the future scope of this study. There has been substantial research to deal with problems like security^63^ and resource allocation^64^ with ML and DL models. As a future scope, with enough models to accurately detect cracks we can form so many use cases to bring it to the consumers.

## Data Availability

The datasets generated and/or analysed during the current study are available in the SDNET2018 repository, https://digitalcommons.usu.edu/cgi/viewcontent.cgi?article=4611&context=cee_facpub^9^, Concrete Crack Images for Classification (CCIC) repository, https://www.kaggle.com/datasets/arnavr10880/concrete-crack-images-for-classification^10^ and Bridge Crack Dataset (BCD) repository, https://www.mdpi.com/2076-3417/9/14/2867^11^.

## References

[1] Yi Y, Zhu D, Guo S, Zhang Z, Shi C (2020). A review on the deterioration and approaches to enhance the durability of concrete in the marine environment. Cement Concr. Compos..

[2] Ham Y, Han KK, Lin JJ, Golparvar-Fard M (2016). Visual monitoring of civil infrastructure systems via camera-equipped unmanned aerial vehicles (UAVs): A review of related works. Vis. Eng..

[3] Sharma KV (2023). Prognostic modeling of polydisperse SiO2/Aqueous glycerol nanofluids’ thermophysical profile using an explainable artificial intelligence (XAI) approach. Eng. Appl. Artif. Intell..

[4] Kanti PK (2023). Thermophysical profile of graphene oxide and MXene hybrid nanofluids for sustainable energy applications: Model prediction with a Bayesian optimized neural network with K-cross fold validation. FlatChem.

[5] Kanti P (2022). Properties of water-based fly ash-copper hybrid nanofluid for solar energy applications: Application of RBF model. Sol. Energy Mater. Sol. Cells.

[6] Kanti PK (2023). The stability and thermophysical properties of Al2O3-graphene oxide hybrid nanofluids for solar energy applications: application of robust autoregressive modern machine learning technique. Sol. Energy Mater. Sol. Cells.

[7] Hsieh YA, Tsai YJ (2020). Machine learning for crack detection: Review and model performance comparison. J. Comput. Civ. Eng..

[8] Munawar HS, Hammad AWA, Haddad A, Soares CAP, Waller ST (2021). Image-based crack detection methods: A review. Infrastructures.

[9] Dorafshan S, Thomas RJ, Maguire M (2018). Sdnet 2018: An annotated image dataset for non-contact concrete crack detection using deep convolutional neural networks. Data Brief.

[10] C¸ a˘glar, F., O^¨^ zgenel, R.: Concrete crack images for classification. Mendeley Data **2** (2019)

[11] Xu H, Su X, Wang Y, Cai H, Cui K, Chen X (2019). Automatic bridge crack detection using a convolutional neural network. Appl. Sci..

[12] Harinath Reddy, C., Mini, K., Radhika, N.: Structural health monitor- ing—an integrated approach for vibration analysis with wireless sensors to steel structure using image processing. In: International Conference on ISMAC in Computational Vision and Bio-Engineering, pp. 1595–1610 (2018). Springer

[13] Pauly, L., Hogg, D., Fuentes, R., Peel, H.: Deeper networks for pavement crack detection. In: Proceedings of the 34th ISARC, pp. 479–485 (2017). IAARC

[14] Lins RG, Givigi SN (2016). Automatic crack detection and measurement based on image analysis. IEEE Trans. Instrum. Meas..

[15] Shahrokhinasab E, Hosseinzadeh N, Monirabbasi A, Torkaman S (2020). Performance of image-based crack detection systems in concrete structures. J. Soft Comput. Civ. Eng..

[16] Munawar HS, Hammad AW, Haddad A, Soares CAP, Waller ST (2021). Image-based crack detection methods: A review. Infrastructures.

[17] Zou Q, Cao Y, Li Q, Mao Q, Wang S (2012). Cracktree: Automatic crack detection from pavement images. Pattern Recognit. Lett..

[18] Salman M, Mathavan S, Kamal K, Rahman M, Salman M, Mathavan S, Kamal K, Rahman M (2013). Pavement crack detection using the Gabor filter. 16th International IEEE Conference on Intelligent Transportation Systems (ITSC 2013).

[19] Niu B, Wu H, Meng Y, Niu B, Wu H, Meng Y (2020). Application of cem algorithm in the field of tunnel crack identification. 2020 IEEE 5th International Conference on Image, Vision and Computing (ICIVC).

[20] Chhabra G, Onyema EM, Kumar S, Goutham M, Mandapati S, Iwendi C (2022). Human emotions recognition, analysis and transformation by the bioenergy field in smart grid using image processing. Electronics.

[21] Baltazart V, Nicolle P, Yang L, Baltazart V, Nicolle P, Yang L (2017). Ongoing tests and improvements of the mps algorithm for the automatic crack detection within grey level pavement images. 2017 25th European Signal Processing Conference (EUSIPCO).

[22] Jo J, Jadidi Z (2020). A high precision crack classification system using multi-layered image processing and deep belief learning. Struct. Infrastruct. Eng..

[23] Landstrom A, Thurley MJ (2012). Morphology-based crack detection for steel slabs. IEEE J. Sel. Top. Signal Process..

[24] Prasanna P, Dana KJ, Gucunski N, Basily BB, La HM, Lim RS, Parvardeh H (2014). Automated crack detection on concrete bridges. IEEE Trans. Autom. Sci. Eng..

[25] Lin M, Zhou R, Yan Q, Xu X, Lin M, Zhou R, Yan Q, Xu X (2019). Automatic pavement crack detection using hmrf-em algorithm. 2019 International Conference on Computer, Information and Telecommunication Systems (CITS).

[26] Pratico FG, Fedele R, Naumov V, Sauer T (2020). Detection and monitoring of bottom-up cracks in road pavement using a machine-learning approach. Algorithms.

[27] Zhang F, Hu Z, Fu Y, Yang K, Wu Q, Feng Z (2020). A new identification method for surface cracks from uav images based on machine learning in coal mining areas. Remote Sens..

[28] Zhang L, Wang Z, Wang L, Zhang Z, Chen X, Meng L (2021). Machine learning-based real-time visible fatigue crack growth detection. Digit. Commun. Netw..

[29] Dharneeshkar J, Aniruthan S, Karthika R, Parameswaran L, Dharneeshkar J (2020). Deep learning based detection of potholes in indian roads using yolo. 2020 International Conference on Inventive Computation Technologies (ICICT).

[30] Li H, Zong J, Nie J, Wu Z, Han H (2021). Pavement crack detection algorithm based on densely connected and deeply supervised network. IEEE Access.

[31] Zhang L, Yang F, Zhang YD, Zhu YJ, Zhang L, Yang F, Zhang YD, Zhu YJ (2016). Road crack detection using deep convolutional neural network. 2016 IEEE International Conference on Image Processing (ICIP).

[32] Meng X (2021). Concrete crack detection algorithm based on deep residual neural networks. Sci. Program..

[33] Su C, Wang W (2020). Concrete cracks detection using convolutional neural- network based on transfer learning. Math. Problems Eng..

[34] Ye X-W, Jin T, Chen P-Y (2019). Structural crack detection using deep learning–based fully convolutional networks. Adv. Struct. Eng..

[35] Feng C, Zhang H, Wang S, Li Y, Wang H, Yan F (2019). Structural damage detection using deep convolutional neural network and transfer learning. KSCE J. Civ. Eng..

[36] Kim CN, Kawamura K, Nakamura H, Tarighat A, Kim CN, Kawamura K, Nakamura H, Tarighat A (2020). Automatic crack detection for concrete infrastructures using image processing and deep learning. IOP Conference Series: Materials Science and Engineering.

[37] Cao M-T, Tran Q-V, Nguyen N-M, Chang K-T (2020). Survey on performance of deep learning models for detecting road damages using multiple dashcam image resources. Adv. Eng. Inform..

[38] Nguyen NHT, Perry S, Bone D, Le HT, Nguyen TT (2021). Two-stage convolutional neural network for road crack detection and segmentation. Expert Syst. Appl..

[39] Park SE, Eem S-H, Jeon H (2020). Concrete crack detection and quantifica- tion using deep learning and structured light. Constr. Build. Mater..

[40] Huyan J, Li W, Tighe S, Xu Z, Zhai J (2020). Cracku-net: A novel deep convolutional neural network for pixelwise pavement crack detection. Struct. Control Health Monit..

[41] Kim B, Yuvaraj N, Sri Preethaa K, Arun Pandian R (2021). Surface crack detection using deep learning with shallow cnn architecture for enhanced computation. Neural Computing Appl..

[42] GI, K.F.: A hierarchical neural network capable of visual pattern recognition. Neural Network **1** (1989).

[43] Russakovsky O, Deng J, Su H, Krause J, Satheesh S, Ma S, Huang Z, Karpathy A, Khosla A, Bernstein M (2015). Imagenet large scale visual recognition challenge. Int. J. Comput. Vis..

[44] LeCun Y (1989). Handwritten digit recognition with a back-propagation network. Adv. Neural Inf. Process. Syst..

[45] Arel I, Rose DC, Karnowski TP (2010). Deep machine learning-a new frontier in artificial intelligence research [research frontier]. IEEE comput. Intel. Mag..

[46] Simonyan, K., Zisserman, A.: Very deep convolutional networks for large- scale image recognition. arXiv preprint arXiv:1409.1556 (2014).

[47] Chollet, F.: Xception: Deep learning with depthwise separable convolutions. In: Proceedings of the IEEE Conference on Computer Vision and Pattern Recognition, pp. 1251–1258 (2017).

[48] He, K., Zhang, X., Ren, S., Sun, J.: Deep residual learning for image recognition. In: Proceedings of the IEEE Conference on Computer Vision and Pattern Recognition, pp. 770–778 (2016).

[49] Szegedy, C., Vanhoucke, V., Ioffe, S., Shlens, J., Wojna, Z.: Rethinking the inception architecture for computer vision. In: Proceedings of the IEEE Conference on Computer Vision and Pattern Recognition, pp. 2818–2826 (2016).

[50] Szegedy, C., Ioffe, S., Vanhoucke, V., Alemi, A.A.: Inception-v4, inception-resnet and the impact of residual connections on learning. In: Thirty-first AAAI Conference on Artificial Intelligence (2017).

[51] Andrew, G. *et al.* Efficient convolutional neural networks for mobile vision applications. Mobilenets. Available: http://arxiv.org/abs/1704.04861 (2017).

[52] Sandler, M., Howard, A., Zhu, M., Zhmoginov, A., Chen, L.-C.: Mobilenetv2: Inverted residuals and linear bottlenecks. In: Proceedings of the IEEE Conference on Computer Vision and Pattern Recognition, pp. 4510–4520 (2018).

[53] Huang, G., Liu, Z., Van Der Maaten, L., Weinberger, K.Q.: Densely connected convolutional networks. In: Proceedings of the IEEE Conference on Computer Vision and Pattern Recognition, pp. 4700–4708 (2017).

[54] Tan M, Le Q, Tan M, Le Q (2019). Efficient Net: Rethinking model scaling for convolutional neural networks. International Conference on Machine Learning.

[55] Sikha O, Bharath B (2022). Vgg16-random fourier hybrid model for masked face recognition. Soft Comput..

[56] Srihari K, Sikha O, Srihari K, Sikha O (2022). Partially supervised image captioning model for urban road views. Intelligent Data Communication Technologies and Internet of Things.

[57] Krishnan G, Sikha O, Krishnan G, Sikha O (2022). Analysis on the Effectiveness of Transfer Learned Features for x-ray Image Retrieval. Innovative Data Communication Technologies and Application.

[58] Brownlee J (2017). A Gentle Introduction to Transfer Learning for Deep Learning.

[59] Wang Y, Zhang JY, Liu JX, Zhang Y, Chen ZP, Li CG, He K, Yan RB (2019). Research on crack detection algorithm of the concrete bridge based on image processing. Proced. Comput. Sci..

[60] Chen C, Seo H, Jun CH, Zhao Y (2022). Pavement crack detection and classification based on fusion feature of LBP and pca with SVM. Int. J. Pavement Eng..

[61] Ojala T, Pietikainen M, Harwood D (1996). A comparative study of texture measures with classification based on featured distributions. Pattern Recognit..

[62] Sari Y, Prakoso PB, Baskara AR, Sari Y, Prakoso PB, Baskara AR (2019). Road crack detection using support vector machine (svm) and otsu algorithm. 2019 6th International Conference on Electric Vehicular Technology (ICEVT).

[63] Shafiq M, Yadav R, Javed AR, Mohsin SAH (2023). CoopGBFS: A Federated Learning and Game-Theoretic Based Approach for Personalized Security, Recommendation in 5G Beyond IoT Environments for Consumer Electronics.

[64] Shafiq M, Tian Z, Liu Y, Aljuhani A, Li Y (2023). ESC&RAO: Enabling seamless connectivity resource allocation in tactile IoT for consumer electronics. IEEE Trans. Consum. Electron..

